# Prescribing Practices in Geriatric Patients with Cardiovascular Diseases

**DOI:** 10.3390/ijerph20010766

**Published:** 2022-12-31

**Authors:** Abdelmoneim Awad, Haya Al-Otaibi, Sara Al-Tamimi

**Affiliations:** 1 Department of Pharmacy Practice, College of Pharmacy, Kuwait University, Kuwait City 13110, Kuwait; 2 Al-Adan Hospital, Ministry of Health, Al-Ahmadi 47005, Kuwait

**Keywords:** prescribing, geriatrics, inappropriate medications, prescribing omissions, cardiovascular diseases, Kuwait

## Abstract

Inappropriate prescribing (IP) increases the risk of adverse medication reactions and hospitalizations in elderly patients. Therefore, it is crucial to evaluate prescribing patterns among this population. This study was designed to assess the prevalence of potentially inappropriate medication (PIMs) use and potential prescribing omissions (PPOs) among geriatrics with cardiovascular diseases (CVDs). In addition, it determined the predictors for IP in this population. A multi-center study was performed retrospectively on 605 patients’ medical records collected randomly from seven governmental hospitals in Kuwait. Three of these hospitals have specialized cardiac centers (tertiary care). Inclusion criteria were age ≥ 65 years, diagnosed with at least one CVD, and attended the outpatient clinic during the last 6 months before data collection. A total of 383 patients (63.3%; 95% CI: 59.3–67.1%) were found using at least one PIM or having PPO or both, based on STOPP/START criteria. Three hundred and ninety-one patients (64.6%; 95% CI: 60.7–68.4%) were prescribed PIMs categorized as C and/or D medicines according to the Euro-FORTA list. Over one-quarter (28.8%; 95% CI: 25.2–32.6%) of the patients had drug–drug interactions class D that require therapy modification and/or X that should be avoided. Patients taking ≥ five medications had significantly higher PIMs based on STOPP and FORTA criteria, drug–drug interactions (*p* < 0.001), and significantly higher PPOs based on START criteria (*p* = 0.041). Patients with three or more chronic diseases had significantly higher PIMs based on STOPP and FORTA criteria and PPOs based on START criteria (*p*-values: 0.028, 0.035, and 0.005, respectively). Significantly higher PIMs based on STOPP criteria and PPOs based on START criteria were found in general hospitals compared to specialized cardiac centers (*p*= 0.002, *p*= 0.01, respectively). These findings highlight the need to develop and implement multifaceted interventions to prevent or minimize inappropriate prescribing among the geriatric population with CVDs in Kuwait.

## 1. Introduction

The elderly population is growing rapidly all over the world. In 2020, the age of 9.3% (727 million) of the global population was ≥ 65 years old, and it is expected to rise to around 16% by 2050 [1]. In Kuwait, the geriatric population increased from 1.8% in 1972 to 3.4% in 2021, growing at an average of 1.33% annual rate. It has been anticipated to increase to 4.41% and 17.9% by 2025 and 2050, respectively. This indicates increased longevity in Kuwait [2]. However, chronic non-communicable illnesses are on the increase and are presently the leading cause of mortality among geriatrics in Kuwait [3]. Cardiovascular diseases (CVDs) are recognized as the major cause of death worldwide; hence, their prevention and management have been designated as a worldwide health priority. In 2019, 17.9 million people died from CVDs, which accounts for 32% of deaths globally. Eighty-five percent of these deaths are caused by heart attack and stroke and two-thirds occurred in patients aged ≥ 70 years [4]. In Kuwait, CVDs account for 41.0% of all deaths [5].

Prescribing medicines is an essential component of elderly care, and its optimization has emerged as a significant public health concern globally. Due to various factors of aging, the prescribing process in the elderly is challenging and complex [6]. These factors comprise the rise in the number of prescribed medicines due to the higher prevalence of chronic illnesses and degenerative diseases, as well as the age-related physiological modifications that have an impact on the pharmacodynamic and pharmacokinetic profiles of the medications. Additionally, manufacturers do not include elderly patients in clinical trials before marketing and there are few literature reports on the use of medications in this population. These factors render the elderly more vulnerable to adverse drug reactions, drug–disease and/or drug–drug interactions, higher rates of hospitalization, poor clinical outcomes, and higher healthcare expenditures [6,7,8]. Polypharmacy is one of these factors that has no universally accepted definition. The present working definitions include ≥4 medications [9] and ≥5 medications, ≥10 medications (hyperpolypharmacy) or ≥ 15 medications (extreme hyperpolypharmacy), as well as qualitative definitions such as “more medications than necessary” [10].

Inappropriate prescribing (IP) is prevalent among elderly patients and includes the utilization of potentially inappropriate medications (PIMs) and/or potential prescribing omissions (PPOs). PIMs are medications whose risk of adverse events outweighs their expected benefits, especially when evidence exists for an effective and safer alternative medicine for the same disease. It also involves the use of a medicine that can cause harmful effects through drug–drug or drug–disease interactions, and the prescribing of medicines at too high a dosage or for a prolonged duration [6,8]. PPOs are clinically indicated medications which are omitted from the patient’s treatment without valid reasons. PIMs and/or PPOs among geriatric patients have been associated with hospital readmissions, high health care expenditures, and mortality [11]. 

Explicit criteria to identify PIMs and/or PPOs are frequently used to evaluate the quality of prescribing among geriatrics, which are useful and offer substantial insights to enhance treatment strategies in healthcare settings [12,13]. These criteria include the American Geriatric Society Beers (AGS) criteria, Screening Tool of Older People’s Potentially Inappropriate Prescriptions criteria and Screening Tool to Alert to Right Treatment (STOPP/START) criteria, and the FORTA (Fit fOR The Aged) list [12,13,14]. 

Several studies were performed using these criteria to indicate the incidence of PIMs/PPOs in older adults with various medical conditions within diverse healthcare settings in various countries globally, mostly in Western countries [7,11,15,16,17,18,19,20,21,22,23,24,25,26,27]. Few studies were reported from the Middle East and North Africa (MENA) region [28,29,30,31]. Only one study was performed in Kuwait to determine PIM use in 420 elderly patients in 10 primary healthcare settings using the Beers, STOPP, FORTA, and Medication Appropriateness Index (MAI) criteria. In addition, START criteria were used to identify the PPOs. Its findings revealed that 53.1%, 55.7%, and 44.3% of the patients were using PIMs based on Beers, STOPP, and FORTA criteria, respectively. According to the MAI criteria, 74% of the patients were found to have one or more inappropriate ratings among their medicines. Almost 20% of the patients had at least one PPO based on START criteria, 19.8% of patients. When using the MAI as a reference standard, STOPP criteria provided the highest sensitivity and measure of agreement to identify PIMs in comparison with Beers and FORTA criteria [31]. Limited literature reported the incidence of IP among older adults with CVDs [32,33,34,35,36,37,38,39]. Only one study was performed in Bahrain to identify IP among geriatrics with hypertension or hypertension and diabetes using STOPP criteria in a primary healthcare setting [36].

CVDs in older adults impose a significant burden in terms of death, morbidity, disability, functional decline, and healthcare expenses [40]. There is a growing interest in providing evidence-based management for patients with CVDs in Kuwait [41]. In addition, due to the rise in the elderly population, there is an increasing demand to enhance their health and quality of life, as well as promote optimal medication prescribing. Nevertheless, there is a paucity of literature evaluating the patterns of prescribing among geriatric patients with CVDs in Kuwait. Therefore, understanding the extent of the problem and the factors that contribute to it is crucial in planning future interventional strategies to prevent IP in geriatric clinical practice and to improve cardiovascular health. The present study was designed to assess the prevalence of PIM use and PPOs in older adults with CVDs attending secondary and tertiary healthcare settings in Kuwait. In addition, it determined the predictors for IP in this population.

## 2. Materials and Methods

### 2.1. Study Design

A multi-center study was conducted to assess the prescribing quality for geriatrics diagnosed with CVDs in secondary and tertiary public healthcare settings in Kuwait. The healthcare system in Kuwait incorporates the public and private sectors, with the former being the main provider. Kuwait is a Middle Eastern country with an area of 17,820 km^2^ and a total population of 4,269,377 individuals [2]. Data were collected retrospectively between March and May 2022. 

### 2.2. Ethical Approval

The Medical Research Ethics Committee of the Ministry of Health in Kuwait granted ethical approval (Ethics No: 2022/150). Informed consent was not required as data were collected from patients’ medical records retrospectively. Data were extracted anonymously as directly identifying patient information was not obtained. 

### 2.3. Inclusion Criteria 

Patients aged 65 years or older with a past medical history of at least one CVD who attended the outpatient cardiology clinics in 3 specialized cardiac centers and 4 general hospitals within the past six months before the date of data collection.

### 2.4. Sample Size and Sampling Strategy

The sample size was calculated by the PS: Power and Sample Size Calculator V.3.1.6 [42]. A minimum sample of 452 patients’ medical records would be required to ascertain a 15% difference in the proportion of PIMs between two groups, for example, secondary care (generalized hospitals) and tertiary care (specialized cardiac centers) with 95% power and a 5% significance level. Assuming that patients’ medical records were incomplete with insufficient information, a sample size of 605 patients’ medical records was acquired (300 from 3 specialized cardiac centers and 305 from 4 general hospitals with cardiology units). Due to the absence of precise information on the number of all patients, including the older adults, who attended the cardiology outpatient clinics at the hospitals during the previous 6 months before the data collection date, it was decided to collect 100 patients’ medical records from each of the three specialized cardiac centers and 75–80 patients’ medical records from each of the four general hospitals.

### 2.5. Data Collection 

Data were acquired through systemic random sampling of the medical records of patients who visited the cardiology outpatients’ clinics during the last 6 months before the data collection date. Data were obtained anonymously since direct information related to patient identification was not obtained. The data collection form comprised three sections. The first section retrieved information regarding the patients’ demographic characteristics (age, gender, nationality, and name of the health facility). The second section contained information about chronic diseases and the latest records of physical measurements (blood pressure and heart rate), laboratory parameters (creatinine, estimated glomerular filtration rate (eGFR), potassium, alanine transaminase, aspartate transaminase, alkaline phosphatase, total bilirubin, albumin, INR, HbA1c, lipid levels) and Child–Pugh score. The third section was designed to obtain information about the prescribed medications (name of the medicine, strength, and dose schedule). Dermatological medications and topical preparations, which include eye drops, intranasal sprays, analgesics, anti-inflammatory creams or gels, and patches, were excluded from the study.

### 2.6. Tools Used for Evaluation of Prescribing Appropriateness 

Three pharmacists who are the authors of the current study independently reviewed and assessed the extracted data. The first author is a clinical professor with expertise in cardiovascular and geriatric pharmacotherapy. Discrepancies were resolved by discussion until a consensus was accomplished. The PIMs and PPOs were identified using the following tools:

#### 2.6.1. STOPP/START Criteria

STOPP criteria contain *“a section related to the indication of medication (a drug prescribed without an evidence-based clinical indication, drug prescribed beyond the recommended duration, where treatment duration is well defined, and duplicate therapy); and other sections in which medications arranged according to a physiological system accompanied by an explanation of why they are potentially inappropriate*”. The three criteria related to the indication of medications and 17 criteria related to cardiovascular and coagulation disorders were used in this study. *“START criteria contain medications arranged according to a physiological system that should be considered for people with certain conditions (potential prescribing omissions [PPOs])”.* Eight of its criteria that are related to cardiovascular and coagulation disorders were used in this study. Therefore, a total of 28 criteria were used in this study to evaluate prescribing appropriateness in elderly patients with CVDs [13].

#### 2.6.2. American Heart Association (AHA) List of Medications That May Cause or Exacerbate Heart Failure

The 2016 AHA list identifies medicines that may cause or worsen heart failure and have a level of evidence of A or B among patients diagnosed with heart failure. Anticancer medications were excluded from the list [43].

#### 2.6.3. FORTA (Fit fOR The Aged) List

The 2018 FORTA list was used to identify class C and D medications, which are considered in the present study as PIMs. FORTA classification system includes 4 classes defined as follows: (1) *Class A (Absolutely): “indispensable drug, clear-cut benefit in terms of efficacy/safety ratio, proven in elderly patients for a given indication”; (2) Class B (Beneficial): “drugs with proven or obvious efficacy in the elderly, but limited extent of the effect and/or safety concerns”; (3) Class C (Careful):” drugs with questionable efficacy/safety profiles in the elderly, which should be avoided or omitted in the presence of too many drugs, absence of benefits or emerging side effects; explore alternatives”; and (4) Class D (Don’t): “avoid if at all possible in the elderly, omit first and use alternative substances”* [14].

#### 2.6.4. Potential Drug–Drug Interactions

Potential drug–drug interactions were recognized by the Lexicomp database [44]. Drug–drug interactions are categorized into 5 groups, which are A, B, C, D, and X. *“Group A indicates no known interaction, B indicates no action needed, C indicates monitor therapy, D indicates consider therapy modification, and X indicates avoid combination.”* [44]. Drug–drug interactions that are classified as D or X were considered in the present study as PIM use.

#### 2.6.5. Appropriateness of Medication Use Based on Renal and Liver Function Tests 

The Lexicomp database was used to determine if each prescribed medication and its scheduled dose were appropriate according to the patient’s latest eGFR, liver function tests, and/or Child–Pugh score [44].

### 2.7. Target Blood Pressure (BP)

The attainment of target BP was assessed according to the 2017 ACC/AHA guidelines [45] and the 2018 ESC/ESH guidelines [46].

### 2.8. Statistical Analysis 

The Statistical Package for Social Sciences (IBM SPSS Statistics for Windows, version 28) was used for data entry and analysis. The results were reported as percentages (95% confidence intervals-CI), medians (interquartile range-IQR), and means (standard deviation-SD). The Shapiro–Wilk and Kolmogorov–Smirnov tests revealed that the continuous data were not normally distributed. The purposeful selection strategy of variables was used for model building to identify the predictors for PIMs based on STOPP criteria, FORTA list, drug–drug interactions, and PPOs based on START criteria. The primary step was to evaluate the unadjusted association between patients’ demographic and clinical characteristics as independent variables (age, gender, nationality, hospitals, number of chronic diseases, and number of medicines) and the outcome variables (PIMs based on STOPP criteria, FORTA list, drug–drug interactions, and PPOs according to START criteria). The dependent/outcome variables were categorized as follows: (1) being prescribed PIM according to STOPP criteria (0 = no, 1 = yes); (2) being prescribed PIM according to FORTA list (0 = no, 1 = yes); (3) being prescribed PIM according to drug–drug interactions (0 = no, 1 = yes); and (4) had PPO according to START criteria (0 = no, 1 = yes). In the multivariable logistic regression analysis, all variables with *p* < 0.25 in the univariate analysis were included to identify the independent variables that are good predictors of the outcome variables. The literature supports a cutoff value of 0.25 [47]. The second step was to fit the multivariable model including all variables identified in the primary step. Independent variables which had no contribution to the model were excluded (for the STOPP criteria model: age, gender, and nationality were excluded with *p*-values of 0.834, 0.610, and 0.920, respectively; for the FORTA list model: age and nationality were excluded with *p*-values of 0.522 and 0.957, respectively; for drug–drug interactions model: nationality, hospitals, and the number of diseases were excluded with *p*-values 0.519, 0.633, and 0.783, respectively; for START criteria: age and gender were excluded with *p*-values 0.786 and 0.775, respectively). The partial likelihood ratio test was used to compare the two models for each outcome variable to ensure that the parsimonious model fit as well as the original model. The coefficients of variables in the parsimonious model were compared to those in the original one. The alteration of coefficients (Δβ) was <15%, indicating that the excluded variables were not crucial in terms of providing a needed adjustment for one or more of the remaining independent variables in the model. In the third step, the results for the potential interactions between the covariates showed that the *p*-values for interaction were far from the level of significance. The last step was to assess the fit of each model using the Hosmer–Lemeshow goodness of fit test. The findings for STOPP criteria model: (chi-squared = 1.775, df = 8, *p*-value = 0.987), FORTA list model (chi-squared = 5.753, df = 8, *p*-value = 0.657), drug–drug interactions model (chi-squared = 2.160, df = 6, *p*-value = 0.984), and START criteria model (chi-squared = 2.479, df = 6, *p*-value = 0.871). Each model’s *p*-value indicated the non-significant difference between the observed and predicted values, demonstrating a good logistic regression model fit. The findings of the multivariable logistic analysis are presented as adjusted odds ratios (AOR) and 95% CI. Statistical significance was accepted at a *p*-value of < 0.05 based on two-sided tests.

## 3. Results

### 3.1. Demographic and Clinical Characteristics of the Patients 

Six hundred and five patients’ medical records were collected for the current study. The median (IQR) age of the patients was 71 (8) [mean (SD) 72.4 (6); range 65–93]. Table 1 presents the demographic and clinical characteristics of the patients. The most common chronic diseases were hypertension (92.1%), ischemic heart disease (77.9%), dyslipidemia (65.0%), and type 2 diabetes (61.8%). The median (IQR) number of chronic diseases was four (2) [mean (SD) 4.4 (2.4)]. Most of the patients had three chronic diseases or more (91.4%). Patients with abnormal renal and liver parameters were as follows: eGFR < 60 mL/min (*n* = 173; 28.6%), serum potassium > 5 mmol/L (9.6%), ALT > 50 IU/L (4.1%), AST > 50 IU/L (44.1%;), ALP > 120 IU/L (38.7%). From the available data for calculating the Child–Pugh score for 213 patients, 6.1% had a Child-Pugh score of class B. Patients with SBP ≥ 130 and/or DBP 80–89 who did not reach the target BP according to the 2017 ACC/AHA guidelines (*n* = 261 out of 446; 58.5%) and according to the 2018 ESC/ESH guidelines with SBP ≥ 140 and/or DBP ≥ 90 (*n* = 184 out of 446; 41.3%). Patients who did not reach the target lipid profile were as follows: LDL-C ≥ 1.81 mmol/L (*n* = 299 out of 537; 55.7%;), non-HDL-C ≥ 2.59 mmol/L (*n* = 258 out of 522; 49.4%), and TG ≥ 1.7 mmol/L (*n* = 143 out of 541; 26.4%), and HbA1c ≥ 7.5% (*n* = 131 out of 327; 40.1%).

Table 2 presents the categories of medications prescribed for the patients. The total number of medications prescribed to the patients was 4584. The most common categories of prescribed medicines were cardiovascular medications (51.9%), anticoagulants and antiplatelets (14.3%;), and antidiabetics (11.1%). The median (IQR) number of prescribed medicines per patient was seven (3) [mean (SD) 7.6 (2.9); range 1–16]. Among the study population, 519 (85.8%) patients were using five or more medications. 

### 3.2. Potentially Inappropriate Medications (PIMs) according to STOPP Criteria 

A total of 383 patients (63.3%; 95% CI: 59.3–67.1) were found using at least one PIM or PPO or both based on STOPP/START criteria. Of these, 103 (26.9%) had at least one PIM, 195 (50.9%) had at least one PPO, and 85 (22.2%) had at least one PIM and one PPO. One hundred and eighty-eight patients (31.1%; 95% CI: 27.4–35.0) were prescribed at least one PIM based on STOPP criteria. Of these patients, 155 (82.5%) had one PIM, 29 (15.4%) had two PIMs, 3 (1.6%) had three PIMs, and 1 (0.5%) had four PIMs. One hundred and ninety-seven out of the 4584 prescribed medicines (4.3%; 95% CI: 3.7–4.5) were PIMs based on STOPP criteria. 

The most prevalent PIM was the use of aspirin plus clopidogrel as secondary stroke prevention for patients who had no coronary stent(s) inserted in the previous year or concurrent ACS (52.4%). The second most common PIM was medication prescribed without any evidence-based clinical indication (*n* = 110; 18.2%). Of these patients, 94 (85.5%) had one medication, 15 (13.6%) had two medications, and 1 (0.9%) had three medications. These medications included antihistamine, betahistine, mebeverine, itopride, orphenadrine/acetaminophen, chlordiazepoxide/clidinium, vitamin B complex, and proton pump inhibitors. The use of ACEIs or ARBs in patients with serum potassium > 5.0 mmol/L (10.0%) was the third most common PIM, followed by thiazide diuretic with serum potassium < 3.0 mmol/L or with a history of gout, aspirin with anticoagulant in patients with chronic atrial fibrillation without ischemic heart disease, centrally-acting antihypertensives without clear intolerance of, or lack of efficacy with, other classes, and duplicate therapy. The duplicate therapies included two proton pump inhibitors, two statins, two beta-blockers, and two nonsteroidal anti-inflammatory medications. Table 3 shows the PIMs according to STOPP criteria.

### 3.3. Potentially Prescribing Omissions (PPOs) according to START Criteria

According to START criteria, 280 patients (46.3%; 95% CI: 42.3–50.4) were with at least one PPO. Of these patients, 207 (73.9%) had one PPO, 50 (17.9%) had two PPOs, 18 (6.4%) had three PPOs, 4 (1.4%) had four PPOs, and 1 (0.4%) had five PPOs. The most prevalent PPOs were antihypertensive therapy where BP > 160/90 mmHg (and BP > 140/90 mmHg, if diabetic) (29.9%), and ACEIs with systolic heart failure and/or coronary artery disease (23.3%). Table 4 presents the PPOs according to START criteria among the patients.

### 3.4. Medications That May Cause or Worsen Heart Failure with a Level of Evidence of A or B 

Of the forty patients with heart failure, four (10.0%; 95% CI: 3.3–24.6%) were prescribed medications that may cause or worsen heart failure with a level of evidence B, according to the AHA statement 2016 [43]. These medications were moxonidine and sitagliptin. 

### 3.5. Potentially Inappropriate Medications (PIMs) according to the FORTA List

Over three-fifths (*n* = 391; 64.6%; 95% CI: 60.7–68.4) of the study population were using PIMs categorized as C (*n* = 343; 87.7%; 95% CI: 84.0–90.7) or D (*n* = 23; 5.9%; 95% CI: 3.9–8.8), or C and D (*n* = 25; 6.4%; 95% CI: 4.3–9.4) medicines according to the FORTA list. Of those patients, 252 (64.5%; 95% CI: 59.5–69.2) were prescribed one PIM, 104 (26.6%; 95% CI: 22.3–31.3) two PIMs, 23 (5.9%; 95% CI: 3.9–8.8) three PIMs, 10 (2.6%; 95% CI: 1.3–4.8) four PIMs, and 2 (0.5%; 95% CI: 0.1–2.0) five PIMs. Five hundred and seventy-nine out of the 4584 prescribed medications (12.6%; 95%CI: 11.7–13.6) were categorized as C or D medicines according to the FORTA list. Of these medicines, 537 (92.7%; 95% CI: 90.2–94.7) were class C and 42 (7.3%; 95% CI: 5.3–9.8) were class D. The most commonly prescribed class C medicines were ascribed to cardiovascular (long-acting nitrates, centrally acting antihypertensive, spironolactone, alpha-blocker, hydralazine, ivabradine, fenofibrate, digoxin, amiodarone), CNS (carbamazepine, duloxetine, amitriptyline, pregabalin, bromazepam, mirtazapine, sertraline, sodium valproate), gastrointestinal (metoclopramide, ranitidine, prucalopride, and antidiabetic (sodium-glucose co-transporter-2 inhibitors and glimepiride). The most common class D medications prescribed were benzodiazepine, phenytoin, serotonin and norepinephrine reuptake inhibitor, chlordiazepoxide/clidinium, glibenclamide, verapamil, sotalol, and nonsteroidal anti-inflammatory drug.

### 3.6. Potential Drug–Drug Interactions

Over one-quarter of patients (*n* = 174; 28.8%, 95% CI: 25.2–32.6%) were taking medications with drug–drug interactions class D, which require therapy modification, or class X, which should be avoided. Of these patients, 116 (66.7%; 95% CI: 59.1–73.5%) had class D drug–drug interactions, 41 (23.5%; 95% CI: 17.62–30.7%) had class X drug–drug interactions, and 17 (9.8%; 95% CI: 6.0–15.4) had both class D and X drug–drug interactions. Of these patients, 118 (67.8%; 95% CI: 60.3–74.6) had one drug–drug interaction, 42 (24.1%; 95% CI: 18.1–31.3) had two drug–drug interactions, 10 (5.8%; 95% CI: 3.0–10.6) had three drug–drug interactions, and 4 (2.3%; 95% CI: 0.74–6.2) had ≥ four drug–drug interactions. Common class D drug–drug interactions included beta-blockers (bisoprolol, metoprolol, carvedilol) + centrally acting antihypertensive agents (moxonidine, methyldopa), nonsteroidal anti-inflammatory drug + aspirin adult low dose or direct oral anticoagulant, clopidogrel + direct oral anticoagulant, atorvastatin + verapamil or phenytoin, simvastatin + lercanidipine or amlodipine or diltiazem, methotrexate + esomeprazole or omeprazole, and warfarin + fenofibrate or Gingko Biloba or allopurinol. Common class X drug–drug interactions included clopidogrel + esomeprazole or omeprazole, carvedilol + fluticasone/formoterol, orphenadrine/acetaminophen + moxonidine or chlordiazepoxide/clidinium, and apixaban + carbamazepine.

### 3.7. Inappropriate Medication Use Based on Renal and Liver Function Tests 

Fourteen (2.3%; 95% CI: 1.3–4.0) patients were using medications with inappropriate dosing based on their recent eGFR. Of these patients, 13 (92.9%) were using one medication and 1 (7.1%) was using three medications. Nine (1.5%; 95% CI: 0.7–2.9) patients were taking medications that are contraindicated based on their recent eGFR. Of these patients, six (66.7%) were taking one medication and three (33.3%) were taking two medications. According to patients’ recent Child–Pugh score and/or liver function tests, two (0.3%; 95% CI: 0.06–1.3) patients were using medications with inappropriate dosing, and five (0.8%; 95% CI: 0.3–2.0) were using contraindicated medications. 

### 3.8. Factors Associated with Inappropriate Prescribing

The univariate analysis revealed that age, gender, and nationality were not significantly associated with PIMs based on STOPP criteria (*p*-values of 0.867, 0.637, 0.277, respectively). Hospitals, the number of diseases, and the number of medications were found to be significantly associated with PIMs according to STOPP criteria (*p*-values of <0.001, 0.034, <0.001, respectively). Age, gender, nationality, and hospitals were not significantly associated with PIMs according to the FORTA criteria (*p*-values of 0.381, 0.158, 0.561, 0.217, respectively). The number of diseases and the number of medications were found to be significantly associated with PIMs based on the FORTA criteria (*p*-values of <0.001 for both). Nationality, gender, hospitals, and the number of diseases were not significantly associated with PIMs based on drug–drug interactions (*p*-values of 0.644, 0.196, 0.312, 0.283, respectively). Age and the number of medications were significantly associated with PIMs due to drug–drug interactions (*p*-values of 0.021, <0.001, respectively). Age, gender, and nationality were not significantly associated with PPOs according to START criteria (*p*-values of 0.904, 0.630, 0.171, respectively). Hospitals, the number of diseases, and the number of medications were significantly associated with PPOs based on START criteria (*p*-values of 0.024, 0.036, 0.044, respectively).

According to the findings of the multivariable logistic regression analysis presented in Table 5, one variable was significantly and independently associated with IP. Patients taking ≥ five medicines had significantly higher PIMs based on STOPP criteria, FORTA criteria, and drug–drug interactions (*p* < 0.001), and significantly higher PPOs according to START criteria (*p* = 0.041), compared with those taking ≤ four medicines. Patients with three or more chronic diseases had significantly higher PIMs based on STOPP and FORTA criteria and PPOs based on START criteria (*p*-values: 0.028, 0.035, and 0.005, respectively), compared with those who had one or two chronic diseases. Significantly higher PIMs based on STOPP criteria and PPOs based on START criteria were found in general hospitals (secondary care) compared to specialized cardiac centers (tertiary care) (*p*-values: 0.002 and 0.01, respectively). 

## 4. Discussion

Based on the literature search, this is the first study to be undertaken in Kuwait and most likely in the MENA region to determine the prevalence of PIM use and PPOs among geriatric patients with CVDs, and to determine the predictors for IP in this population. In addition, it is the second literature report from Kuwait to indicate the prevalence of PIMs and PPOs among geriatric patients. The previous study reported the PIMs and PPOs among 420 geriatric patients with or without CVDs in 10 primary healthcare settings according to Beers, STOPP/START, FORTA, and MAI criteria [31]. In the present study, five screening tools were used simultaneously among 605 geriatric patients with CVDs attending secondary and tertiary healthcare settings to identify IP. The current results could be the primary step toward offering baseline data on potentially IP in elderly patients with CVDs, which would be useful in the development and assessment of future interventions to improve medication use among geriatrics and ensure their efficacy and safety. In addition, they enable vital comparative work with the available and forthcoming similar research within the MENA region, and globally.

The present results revealed that 31.1% of patients with CVDs were prescribed at least one PIM based on STOPP criteria, compared to previous reports among patients with hypertension from Bahrain (34.1%) and Spain (27.8%) [33,36]. According to the STOPP/START criteria, the prevalence of patients with PIMs and/or PPOs in the current study was 63.3%, which is higher than the prevalence reported in two Ethiopian studies (37.2% and 61.5%). This might be attributed to the greater prevalence of polypharmacy among the patients in this study, where the mean (SD) number of prescribed medications was 7.6 (2.9) compared to 4.92 (1.86) and 3.97 (1.55) in those studies [32,35]. The most prevalent PIM in the present study was the use of aspirin plus clopidogrel as secondary stroke prevention for patients who had no coronary stent(s) inserted in the previous year or concurrent ACS, which was the second PIM in Getachew et al.’s 2016 study and not present in Abegaz et al.’s 2018 study [32,35]. In the two Ethiopian reports, the most common PIM was the use of aspirin with anticoagulant in patients with chronic atrial fibrillation without ischemic heart disease, which was the fifth in our study. This IP of anticoagulants for geriatric patients could escalate the tendency of gastrointestinal bleeding [13]. The second prevalent PIM was medication prescribed without any evidence-based clinical indication, which was the second in Abegaz et al.’s 2018 study [35] and the fourth in Getachew et al.’s 2016 study [32]. This will increase the risk for inappropriate polypharmacy, which renders older adults more susceptible to adverse medication events along with drug–disease and/or drug–drug interactions, and non-adherence [6,7]. According to the FORTA list, 64.6% of the study population were taking PIMs categorized as C and/or D medications, which is greater than the rates indicated in a study conducted among elderly patients with and without CVDs attending primary healthcare settings in Kuwait (44.3%) and hospitalized geriatric patients in Germany (58.0%) [27,31]. The present findings show that only four patients (10.0%) out of 40 with heart failure were using medicines that may cause or worsen heart failure, which is lower than two studies performed in the USA (47%) among 221 geriatric patients with HFpEF [48], and 11.9% in 8993 patients with HFrEF [49]. Differences within the study populations could account for this, as in our study, data were collected from patients with different CVDs, whereas those studies included only patients with heart failure. Over one-quarter (28.8%) of the patients were taking medications with potential drug–drug interactions, and 2.6% were using medications with inappropriate dosage, which were lower than the rates reported by a Canadian study conducted in geriatric patients of 33.9% and 9.6%, respectively [15]. These findings underscore that these patients are at risk of health hazards, which can lead to emergency visits, hospitalizations, and an increase in healthcare expenditures [6,7,8]. Comparisons of current results to the preceding literature must be approached with caution due to potential sources of variation. These include differences in methodology, such as prospective or retrospective, duration of data collection, medicine availability, healthcare settings, and patient care (hospitalized or outpatients). 

The IP practices revealed by the present study could be explained in part by polypharmacy, which has been found a significant factor associated with the use of PIMs in the study population. Other possible factors may include a lack of comprehensive assessments of medications in geriatrics, clinicians’ knowledge about the hazards of prescribing PIMs, a lack of continuing professional education programs addressing this issue, and the absence of geriatric consultants. In accordance with previously published literature, our study revealed polypharmacy as a significant independent predictor for increased IP [16,17,21,23,24,25,28,29]. Age and gender were stated in the literature as predictors for IP [7,16,17,20,24], even though neither of these two variables was found to be a predictor for IP in the current study, which is in agreement with previous literature reports [23,25]. This study demonstrated significantly more PIMs and PPOs in general hospitals (secondary care) compared to specialized cardiac centers (tertiary care), which might be attributed to variances in the clinicians’ credentials, clinical practice, and patient health status. The present result is consistent with a prior study, which reported that the use of evidence-based medicines in patients with CVDs was more common among patients whose healthcare providers were cardiologists [50], as is the case in Kuwaiti’s tertiary care, where physicians in secondary care may be internists [51]. Furthermore, it has been demonstrated that the health benefits of subspecialty care for patients with CVD substantially outweigh general care and lead to a decrease in hospital stay, cardiovascular readmission, and death [52]. 

According to START criteria without valid contraindications, 46.3% of patients in the current study had one or more clinically indicated medications omitted from their treatment, which is higher than in Spain (35.0%) and Ethiopia (21.2%) [32,33]. The commonly prevalent PPOs according to START criteria were antihypertensive therapy where BP > 160/90 mmHg (and BP > 140/90 mmHg, if diabetic) followed by ACEIs with systolic heart failure and/or coronary artery disease, while in the Ethiopian report, the first PPO was ACEIs with systolic heart failure and/or coronary artery disease, followed by antihypertensive therapy where BP > 160/90 mmHg [35]. One possible reason for these PPOs is that some physicians do not follow clinical practice guidelines and favor treating patients based on clinical experience. Further possible explanations include clinicians’ desire to avoid polypharmacy due to concerns about adverse effects and non-adherence when prescribing more medicines to the patient. The latter could be a reasonable cause for PPOs, as the present study demonstrated that polypharmacy was significantly associated with PPOs. In addition, this study revealed that having three or more chronic diseases was a predictor for PPOs, which may discourage clinicians from the addition of clinically needed medications.

The present study showed incomplete documentation of patients’ medication records regarding patients’ BP and laboratory results, which is in line with a prior study performed in Kuwait [51]. This might be due to habit or a busy practice schedule and time restraints. The majority of clinicians may devote their limited time to offering patient care, making documentation a secondary priority. The current study revealed low attainment of optimal therapeutic targets for BP, LDL-C, non-HDL-C, and HbA1c among the patients, which are close to the findings of an earlier study performed in Kuwait [51]. This is concerning and elevates the risk for recurring cardiovascular events, since the effective treatment of CVDs entails not only managing a specific condition but also treating and preventing CVD risk factors such as dyslipidemia, diabetes, and hypertension. According to the literature, possible causes of not achieving target therapeutic goals include IP patterns, poor medication adherence, and a lack of awareness of the significance of achieving optimal therapy goals [53]. These findings may assist in offering baseline information on those who have not met their target therapeutic goals, which could be utilized to plan and evaluate any forthcoming interventions or initiatives for improving therapeutic goal attainment among this population. 

### 4.1. Implications for Practice and Research

The current results underscore the need to implement multifaceted interventions which are demonstrated to be effective in the literature, such as continuous professional education to improve the knowledge and skills of physicians and pharmacists in geriatric care, a computer decision-support system for electronic prescribing with alerts for PIMs, review of medications by pharmacists, and a multidisciplinary team-based approach [6,18,26,54,55,56]. Geriatric cardiology which integrates adult cardiology and geriatric medicine is an emerging cardiology subspecialty. Its nature can embrace a multidisciplinary team for addressing the elderly-specific features, such as co-morbidities, polypharmacy, functional disability, cognitive decline, frailty, and shared decision-making, which may have an impact on routine cardiovascular care [57]. Therefore, the establishment of an effective multidisciplinary team setting can support the busy cardiovascular physician by providing an extensive range of clinical services to better address geriatric patients with multimorbidity. The role of pharmacists in this team should be specified and reinforced to review, monitor, and optimize drug therapy. Pharmacists have become essential members of the multidisciplinary team through delivering comprehensive clinical services [18,54]. 

Considering the detrimental impact of PIMs and PPOs on elderly patients, future research is necessary to evaluate the effects of IP revealed by the present study on clinical outcomes including medicine-related adverse events, hospitalizations, and death in geriatric patients in Kuwait. The IP practices revealed by this study emphasize the importance of further research to determine the extent of PIM awareness among physicians and pharmacists in healthcare facilities. The present study showed incomplete documentation of patients’ medication records, which emphasizes the importance of conducting qualitative research to reveal the causes for this inadequate documentation.

### 4.2. Strengths and Limitations of the Study

This study’s strengths are (i) appropriate sample size and sampling method that generated data from a representative sample of the study population; (ii) data from patients’ medical records indicated IP more precisely than data from patient surveys or pharmacy dispensing records; (iii) the first study to determine the prevalence of PIM use and PPOs in elderly patients with CVDs using five screening tools applied concurrently to one group of patients to overcome numerous limitations of utilizing only one tool, and (iv) it adds to the limited literature on prescribing patterns for geriatric patients with CVDs in developing countries, and enables crucial comparative work with the available and forthcoming similar research within the MENA region and globally. This study had certain limitations as potential confounders, including (i) it did not include interviews with physicians to determine the causes for the revealed IP, as well as whether they were aware of prescribing PIMs or monitoring patients for PIM-related adverse events; (ii) it did not evaluate the effect of IP on clinical outcomes, which would have been a valuable addition to the study; and (iii) the study’s retrospective design, which depends on the quality of documentation in the patients’ medical records; the findings should be confirmed with a prospective assessment. 

## 5. Conclusions

The current study reveals IP patterns in older adults with CVDs within secondary and tertiary healthcare settings in Kuwait. Considering the rising proportion of older adults, the prevalence of IP practices is a significant public health challenge. The current results could be the primary step toward offering baseline data on potentially IP among geriatric patients with CVDs, and underscore the need for effective multifaceted intervention strategies that could be directed at the identified areas to optimize prescribing quality, reduce inappropriate polypharmacy, and improve the medication regimen’s appropriateness. Future research is necessary to evaluate the impact of the present study IP rates on clinical outcomes, such as adverse drug events, hospitalizations, and death in geriatrics with CVDs.

## Figures and Tables

**Table 1:** Demographic and clinical characteristics of the patients (*n* = 605).

Characteristics	Frequency (%)
Age (Years)	
65–74	412 (68.1)
75–84	163 (26.9)
≥85	30 (5.0)
Gender	
Male	324 (53.6)
Female	281 (46.4)
Nationality	
Kuwaiti	430 (71.1)
Non-Kuwaiti	175 (28.9)
Hypertension	557 (92.1)
Ischemic heart disease	471 (77.9)
Dyslipidemia	393 (65.0)
Type 2 diabetes	374 (61.8)
Atrial fibrillation	145 (24.0)
Thyroid disease	82 (13.6)
Hypothyroidism	75 (12.4)
Hyperthyroidism	7 (1.2)
Chronic kidney disease	78 (12.9)
Stage 2	4 (0.7)
Stage 3 A	20 (3.3)
Stage 3 B	25 (4.1)
Stage 4	19 (3.1)
Stage 5	10 (1.7)
Asthma	59 (9.8)
Peptic ulcer disease	49 (8.1)
Previous stroke	42 (6.9)
Arthritis	41 (6.8)
Osteoarthritis	26 (4.3)
Rheumatoid Arthritis	15 (2.5)
Chronic heart failure	40 (6.6)
HFpEF	9 (1.5)
HFrEF	16 (2.6)
HFmrEF	4 (0.7)
Missing	11 (1.8)
Benign prostatic hyperplasia	30 (5.0)
Hyperuricemia/Gouty Arthritis	28 (4.6)
Chronic obstructive pulmonary disease	23 (3.8)
Osteoporosis	14 (2.3)
Depression	13 (2.1)
Irritable bowel syndrome	13 (2.1)
Epilepsy	10 (1.7)
Peripheral neuropathy	10 (1.7)
Peripheral arterial disease	8 (1.3)
Urinary incontinence	8 (1.3)
Dementia	6 (1.0)
Cancer	6 (1.0)
Insomnia	4 (0.7)
Psychiatric disorder	3 (0.5)
Parkinson’s disease	2 (0.3)
Number of Diseases	
1–2	52 (8.6%)
≥3	553 (91.4%)
3–5	449 (74.2%)
≥6	104 (17.2%)
Blood Pressure Categories (mmHg)	
*SBP < 120 and/or DBP < 60	86 (14.2)
SBP 120–129 and DBP 70–79	99 (16.4)
SBP ≥ 130 and/or DBP 80–89	77 (12.7)
SBP ≥ 140 and/or DBP ≥ 90	184 (30.4)
Missing	159 (26.3)
Estimated Glomerular Filtration Rate [eGFR] (mL/min)	
≥90	157 (26.0)
60–89	273 (45.1)
45–59 (Stage 3A *CKD)	95 (15.7)
30–44 (Stage 3B CKD	48 (7.9)
15–29 (Stage 4 CKD)	19 (3.1)
<15 (Stage 5 CKD)	11 (1.8)
Missing	2 (0.3)
Serum Potassium (mmol/L)	
<3.5	10 (1.6)
3.5–5.0	530 (87.6)
>5.0	58 (9.6)
Missing	7 (1.2)
Serum Alanine Transaminase [ALT] (IU/L)	
3–50	561 (92.7)
>50	25 (4.1)
Missing	19 (3.1)
Serum Aspartate Transaminase [AST] (IU/L)	
1–50	508 (84.0)
>50	25 (4.1)
Missing	72 (11.9)
Serum Alkaline Phosphatase [ALP] (IU/L)	
30–120	286 (47.3)
>120	234 (38.7)
Missing	85 (14.0)
Child–Pugh Score	
5–6 (Class A)	200 (33.1)
7–9 Class (B)	13 (2.1)
10–15 (Class C)	0 (0)
Missing	392 (64.8%)
Serum low-density lipoprotein cholesterol [LDL-C] (mmol/L)	
<1.4	116 (19.2)
<1.81	122 (20.2)
1.81–2.58	259 (42.8)
≥2.59	40 (6.6)
Missing	68 (11.2)
Serum non-high-density lipoprotein [Non-HDL-C] (mmol/L)	
<2.2	162 (26.8)
<2.59	102 (16.9)
2.59–3.35	163 (26.9)
≥3.36	95 (15.7)
Missing	83 (13.7)
Serum Triglycerides (mmol/L)	
<1.7	398 (65.8)
≥1.7	143 (23.6)
Missing	64 (10.6)
*HbA1c (%)	
<7.5	196 (32.4)
7.5–7.9	36 (5.9)
8.0–8.4	56 (9.3)
≥8.5	39 (6.4)
Missing	278 (46.0)

* HFpEF (Heart failure preserved ejection fraction); HFrEF (Heart failure reduced ejection fraction); HFmrEF (Heart failure with mildly reduced ejection fraction); SBP (Systolic blood pressure); DBP (Diastolic blood pressure); CKD (chronic kidney disease); HbA1c (Hemoglobin A1c).

**Table 2:** Categories of prescribed medications to patients (*n* = 4584).

Categories of Medications	Frequency (%)	95% CI
Cardiovascular	2379 (51.9)	50.4–53.4
Anticoagulants and antiplatelets	657 (14.3)	13.3–15.4
Antidiabetic	511 (11.1)	10.3–12.1
Gastrointestinal	481 (10.5)	9.6–11.4
Vitamins, minerals, and dietary supplements	143 (3.1)	2.6–3.7
Respiratory	79 (1.7)	1.4–2.2
Endocrine	63 (1.4)	1.1–1.8
Central nervous system	56 (1.2)	0.9–1.6
Analgesic and non-steroidal anti-inflammatory	43 (0.9)	0.7–1.3
Benign prostatic hyperplasia	40 (0.9)	0.6–1.2
Gout	30 (0.7)	0.5–1.0
Others (such as skeletal muscle relaxants, bisphosphonates, disease-modifying anti-rheumatic drugs, phosphate binders, urinary incontinence medications, erythropoietin stimulating agents, antihistamines)	102 (2.2)	1.8–2.7
Number of Medications		
1–4	86 (14.2)	11.6–17.3
5–9	375 (62.0)	58.0–65.9
≥10	144 (23.8)	20.5–27.4

**Table 3:** Potentially inappropriate medications (PIMs) based on STOPP criteria among patients.

PIMs	Frequency (%; 95% CI)
1. Any drug prescribed without any evidence-based clinical indication (*n = 605)	110 (18.2; 15.2–21.5)
2. Any drug prescribed beyond the recommended duration (*n* = 605)	0
3. Any duplicate medication (*n* = 605)	17 (2.8; 1.7–4.6)
4. Digoxin for heart failure with normal systolic ventricular function (*n* = 9)	0 (0)
5. Verapamil or diltiazem with class III or IV heart failure (*n* = 16)	0 (0)
6. Beta-blocker with verapamil or diltiazem (*n* = 429)	2 (0.5; 0.06–1.7)
7. Beta-blocker with bradycardia (<50/min) (*n* = 429)	0
8. Amiodarone as first-line antiarrhythmic therapy in supraventricular tachyarrhythmias (*n* = 145)	0
9. Loop diuretic as first-line treatment for hypertension (*n* = 557)	0
10. Loop diuretic for dependent ankle oedema without evidence of heart failure, liver failure, nephrotic syndrome, or renal failure (*n* = 523)	0
11. Loop diuretic for the treatment of hypertension with urinary incontinence (*n* = 8)	0
12. Thiazide diuretic with hypokalemia (sK < 3.0 mmol/L) or with a history of gout (*n* = 119)	10 (8.4; 3.4–13.4)
13. Centrally acting antihypertensives unless clear intolerance or lack of efficacy with other classes (*n* = 557)	18 (3.2; 2.0–5.2)
14. ACEIs or ARBs in patients with hyperkalemia (sK > 5.0 mmol/L) (*n* = 422)	42 (10.0; 7.3–13.2)
15. Spironolactone, or eplerenone with K serving drugs (e.g., ACEI, ARB, Amiloride, Triamterene) without monitoring of sK (*n* = 57)	0
16. Sildenafil or Tadalafil, or Vardenafil in severe HF or concurrent nitrate therapy for angina (*n*= 219)	0
17.Aspirin at doses > 160 mg per day (*n* = 361)	0
18. Aspirin with a history of peptic ulcer disease without concomitant proton pump inhibitor (*n* = 49)	0
19. Aspirin with anticoagulant in patients with chronic atrial fibrillation (No ischemic heart disease) (*n* = 62)	5 (8.1; 3.0–18.6)
20. Aspirin plus clopidogrel as secondary stroke prevention, unless the patient has a coronary stent(s) inserted in the previous 12 months or concurrent ACS (*n*= 42)	22 (52.4; 36.6–67.7)

* *n*: Number of patients in whom the criteria is applicable for assessment.

**Table 4:** Potentially prescribing omissions (PPOs) based on START criteria among patients.

PPOs	Frequency (%; 95% CI)
1. Anticoagulant in the presence of chronic atrial fibrillation (*n* = 145)	16 (11.0; 6.6–17.6)
2. Aspirin in the presence of chronic atrial fibrillation, if anticoagulants are contraindicated (*n* = 145)	0 (0)
3. Antiplatelet therapy with a documented history of coronary, cerebral, or peripheral vascular disease (*n* = 485)	88 (18.1; 14.9–21.9)
4. Antihypertensive therapy where BP > 160/90 mmHg (and BP > 140/90, if diabetic) (*n* = 184)	55 (29.9; 23.5–37.1)
5. Statin therapy with a documented history of coronary, cerebral, or peripheral vascular disease, unless the patient’s status is end-of-life or age is >85 years (*n* = 485)	36 (7.4; 5.3–10.2)
6. ACEIs with systolic heart failure and/or coronary artery disease (*n* = 459)	107 (23.3; 19.6–27.5)
7. Beta-blocker with ischemic heart disease (*n* = 471)	79 (16.8; 13.6–20.5)
8. Appropriate beta-blocker (bisoprolol, nebivolol, metoprolol succinate, or carvedilol) with stable systolic heart failure (*n* = 16)	1 (6.3; 0.3–32.3)

**Table 5:** Association between patients with PIMs based on STOPP, FORTA, and drug–drug interactions, and PPOs based on START criteria, and their characteristics.

Characteristics	STOPP Criteria		FORTA Criteria		Drug–Drug Interactions		START Criteria	
	Adjusted OR (95% CI)	*p*-Value	Adjusted OR (95% CI)	*p*-Value	Adjusted OR (95% CI)	*p*-Value	Adjusted OR (95% CI)	*p*-Value
**Age (Years)** 65–74 75–84 ≥85	-	-	-	-	Reference 0.4 (0.1–1.2) 0.6 (0.2–2.1)	0.111	-	-
**Gender** Male Female	-	-	Reference 1.2 (0.8–1.8)	0.241	Reference 1.4 (0.9–2.0)	0.117	-	-
**Nationality** Kuwaiti Non-Kuwaiti	-	-	-	-	-	-	Reference 1.2 (0.8–1.8)	0.283
**Hospitals** Tertiary care Secondary care	Reference 1.8 (1.2–2.7)	0.002 *	Reference 0.8 (0.5–1.13)	0.180	-	-	Reference 1.6 (1.1–2.2)	0.01 *
**Number of Diseases** 1–2 3–5 ≥6	Reference 2.5 (1.1–5.9) 2.1 (1.2–3.3)	0.028 *	Reference 2.9 (1.3–6.8) 1.9 (1.1–3.4)	0.035 *	-	-	Reference 3.4 (1.6–7.4) 1.8 (1.1–2.8)	0.005 *
**Number of Medications** 1–4 5–9 ≥10	Reference 6.8 (3.2–14.6) 3.5 (2.3–5.4)	<0.001 *	Reference 22.4 (10.0–50.2) 6.2 (3.2–11.7)	<0.001 *	Reference 33.6 (11.6–97.1) 5.8 (3.8–8.8)	<0.001 *	Reference 1.8 (1.0–3.3) 1.5 (1.1–2.3)	0.041 *

- Excluded variables with *p* > 0.25, which were not included in the multivariable analysis; * *p* < 0.05.

## Data Availability

Available from the corresponding author upon request.
